# Effective infection prevention and control measures in long-term care facilities in non-outbreak and outbreak settings: a systematic literature review

**DOI:** 10.1186/s13756-023-01318-9

**Published:** 2023-10-18

**Authors:** Nando Bloch, Jasmin Männer, Céline Gardiol, Philipp Kohler, Jacqueline Kuhn, Thomas Münzer, Matthias Schlegel, Stefan P. Kuster, Domenica Flury

**Affiliations:** 1 Division of Infectious Diseases and Hospital Epidemiology, Cantonal Hospital St.Gallen, St.Gallen, Switzerland; 2 Federal Office of Public Health, Bern, Switzerland; 3 Geriatrische Klinik St.Gallen, St.Gallen, Switzerland

**Keywords:** Infection prevention, Long-term care facilities, Healthcare-associated infection, COVID-19

## Abstract

**Background:**

Healthcare-associated infections in long-term care are associated with substantial morbidity and mortality. While infection prevention and control (IPC) guidelines are well-defined in the acute care setting, evidence of effectiveness for long-term care facilities (LTCF) is missing. We therefore performed a systematic literature review to examine the effect of IPC measures in the long-term care setting.

**Methods:**

We systematically searched PubMed and Cochrane libraries for articles evaluating the effect of IPC measures in the LTCF setting since 2017, as earlier reviews on this topic covered the timeframe up to this date. Cross-referenced studies from identified articles and from mentioned earlier reviews were also evaluated. We included randomized-controlled trials, quasi-experimental, observational studies, and outbreak reports. The included studies were analyzed regarding study design, type of intervention, description of intervention, outcomes and quality. We distinguished between non-outbreak and outbreak settings.

**Results:**

We included 74 studies, 34 (46%) in the non-outbreak setting and 40 (54%) in the outbreak setting. The most commonly studied interventions in the non-outbreak setting included the effect of hand hygiene (N = 10), oral hygiene (N = 6), antimicrobial stewardship (N = 4), vaccination of residents (N = 3), education (N = 2) as well as IPC bundles (N = 7). All but one study assessing hand hygiene interventions reported a reduction of infection rates. Further successful interventions were oral hygiene (N = 6) and vaccination of residents (N = 3). In outbreak settings, studies mostly focused on the effects of IPC bundles (N = 24) or mass testing (N = 11). In most of the studies evaluating an IPC bundle, containment of the outbreak was reported. Overall, only four articles (5.4%) were rated as high quality.

**Conclusion:**

In the non-outbreak setting in LTCF, especially hand hygiene and oral hygiene have a beneficial effect on infection rates. In contrast, IPC bundles, as well as mass testing seem to be promising in an outbreak setting.

**Supplementary Information:**

The online version contains supplementary material available at 10.1186/s13756-023-01318-9.

## Background

In the United States, there are 65,600 regulated long-term care facilities (LTCF). Around 70% of people turning 65 are expected to need long-term care at some point in their life, and 18% of the older persons will spend over a year in a nursing facility [1]. Similar data exist for Europe, where approximately 3 million long-term care beds exist in nursing and residential care facilities in the 26 EU member states for which data are available in 2020 [2].

Healthcare-associated infections (HAI) are a major threat in acute and long-term care [3]. Point prevalence studies from Switzerland demonstrated that between 2.0 and 4.4% of nursing home residents are affected by HAI [4]. In combination, these numbers indicate that a large proportion of the population will sooner or later be affected by HAI in a long-term care institution and that there is an essential need for effective HAI preventive and control measures in these settings [3]. The Covid-19 pandemic underlined the strong need for recommendations to prevent HAI in long-term care [5].

While infection prevention and control (IPC) measures and outcomes are well defined for acute care hospitals in the World Health Organization (WHO) core components for infection prevention [6], data are scarce for long-term care settings.

In a thorough review by Lee et al., published 2019 prior to the Covid-19 pandemic, the authors were unable to identify a set of measures that could be proposed for implementation of effective IPC measures [7]. Up to this review, only a few high-quality studies were available [7].

In the current study, we aimed to both, update the findings by Lee et al. and complete by focusing on the Covid-19 pandemic in order to provide an overview of the current literature, identify existing research gaps and propose IPC measures and that could uniformly be recommended in long-term care. For the analysis, we differentiated between non-outbreak and outbreak settings.

## Methods

The methods and results are reported according to the Preferred Reporting Items for Systematic Review and Meta-analyses (PRISMA) statement 2020 [8].

### Definitions

#### PICOS statement

The *population of interest* was defined as residents and healthcare workers in adult LTCF. *Interventions* included any IPC measures in accordance with the WHO core components for infection prevention even if they were mainly developed for acute care settings [9]. Furthermore, we included oral hygiene as IPC measure as it has been shown to have a beneficial effect on infection rates in other settings [10]. No restrictions in terms of *comparisons* were made. *Outcomes* were defined as HAIs or HAI prevention measures, mortality or transmission events, as well as healthcare worker attributes such as IPC knowledge or adherence to measures.

### Search strategy

In order to cover the most recent scientific evidence, with a specific focus on the Covid-19 pandemic, we performed an electronic search of PubMed and The Cochrane Central Register of Controlled Trials (CENTRAL) using the terms (((infection[Title/Abstract] OR infections[Title/Abstract]) AND (‘nursing home*’[Title/Abstract] OR ‘skilled nursing*’[Title/Abstract] OR ‘long-term care’[Title/Abstract])) AND (practice[Title/Abstract] OR control*[Title/Abstract] OR measure*[Title/Abstract] OR evaluate*[Title/Abstract] OR effect*[Title/Abstract] OR prevent*[Title/Abstract] OR program*[Title/Abstract] OR intervention*[Title/Abstract] OR outcome*[Title/Abstract])) NOT (surgery[Title/Abstract] OR cancer[Title/Abstract] OR ‘neoplasm’[Title/Abstract] OR ‘intensive care unit’[Title/Abstract] OR child[Title/Abstract] OR children[Title/Abstract] OR ‘operative’[Title/Abstract]). Thereby, we built on the search strategy used in the most comprehensive existing review [7], but extended the time frame from 2017 until the 4th of November, 2022. In addition, reference lists of reviewed articles were scanned and the results combined.

### Eligibility criteria

We included randomized controlled trials, observational studies (cohort and case–control studies) and quasi-experimental studies (before-after studies) in non-outbreak settings and outbreak reports. Studies were included if they were published in English and reported results from an infection prevention intervention in adult LTCFs.

Article types such as review papers, letters, editorials, expert opinions, ecological studies and study protocols were excluded, as were studies from pediatric long-term-care settings.

### Study selection

Four main authors (NB, DF, SPK, and JM) screened searched titles and abstracts of each reference identified by the search. If the study met the eligibility criteria, the full-text article was reviewed independently for definitive inclusion by two authors each. In case of disagreement or in unclear cases, a third author made the decision about final inclusion.

### Data extraction

Study data were extracted by the same authors (NB, DF, SPK, and JM), including setting, study design, main topic, type of intervention, and outcomes, using a standardized data collection form. An intervention was rated as successful when a statistically significant effect in the primary outcome was observed.

Included studies were further classified into non-outbreak versus outbreak settings.

### Quality assessment

To assess methodological quality and risk of bias, we used the Cochrane risk-of-bias (RoB) 2.0 tool for randomized controlled trials, and the Newcastle Ottawa Quality Assessment Scale for Cohort studies and case–control studies [11, 12]. Each included study was assessed by one author and classified as high, medium, or low quality.

If the judgement in all key domains was ‘low risk of bias’ for RCT or achieved one star within every category for observational studies, the study was determined to be high quality. If the judgement in one or more key domains was ‘unclear’ or had ‘some risk of bias’ in the RoB 2.0 tool or achieved most but not all stars in the Newcastle–Ottawa-Scale, the study was evaluated to be medium quality. If the study was assessed to be at high risk of bias in one or more key domains for RCTs or failed to meet most of the stars for observational studies, the quality-summary was deemed to be low in quality. Single-arm trials and outbreak reports were classified as low quality.

In order to avoid duplication and for better readability, most results are either presented in the detailed tables or in the main text.

Detailed descriptions of the respective investigated infection control and prevention measures are given in Tables 1 and 2.

## Results

### Study characteristics

**Fig. 1:**
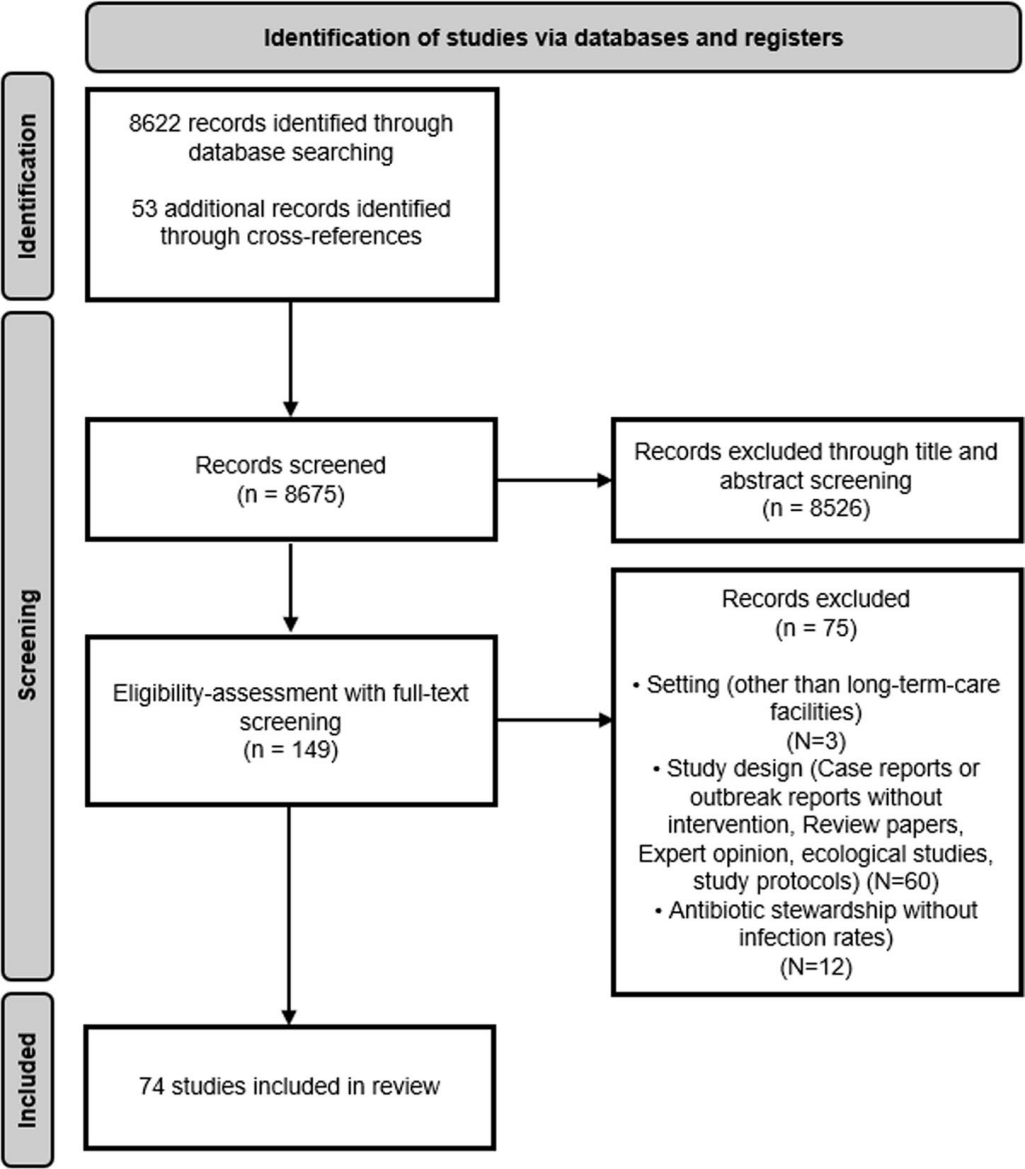
PRISMA flow diagram 2020. Preferred Reporting Items for Systematic Reviews and Meta-Analyses: the PRISMA statement [8]

The literature search yielded 8675 references (Fig. 1). After the screening of titles and abstracts, we selected 150 studies for full-text screening. Seventy-four studies met the inclusion criteria and were included [13–86] (Tables 1, 2).

**Table 1:** Included studies from the non-outbreak setting

Author	Design	Setting	Sample size	Topic	Intervention	Study period	Outcome	Results	Mean quality score
Chahine et al. (2022) [13]	Quasi-experimental	LTCF	205 (2015/16) and 253 (218/19) hospital admissions	Antimicrobial Stewardship	AMS mandate consisting of leadership, accountability, drug expertise, acting, tracking, reporting and education	2015/16 and 2018/19	MDRO and CDI-Incidence	No statistically significant difference in the combined rate of LTCF-acquired MDRO-I/C and CDI	Medium
Felsen et al. (2020) [14]	Quasi-experimental	6 NHs in the USA	*Not described*	Antimicrobial Stewardship	CDC's core elements for antibiotic stewardship in acute care	2014–2019	CDI incidence	Rate of CDI per 10.000 RD decreased	Low
Nace et al. (2020) [15]	RCT	25 LTCFs in the USA	Intervention: 512.408 facility resident-days Control: 443.912 facility resident-days	Antimicrobial Stewardship	Multifaceted antimicrobial stewardship intervention, education, guidelines, audit, feedback	02/2017–04/2018	CDI incidence	Increase in CDI in control group	Medium
Salem-Schatz et al. (2020) [16]	Quasi-experimental	30 LTCFs in the USA	365.019 patient days in first period 340.468 resident days in second period	Antimicrobial Stewardship	Education, tools	1. period: 13/2012–06/2013 2. Period: 11/2013–06/2014	CDI incidence rate	Reduction of CDI	Low
Mody et al. (2003) [17]	RCT	2 LTCFs in the USA	127 persistent carriers	Decolonization	Mupirocin therapy or placebo administered twice daily for 14 days to nares and/or wound surfaces	Not reported	*S.aureus* colonization, reduction in *S.aureus* infections in residents treated with Mupirocin	Mupirocin significantly eradicated colonization in 93% of intervention group while 85% of placebo group remained colonized	Medium
Baldwin et al. (2010) [19]	cRCT	32 NHs in Northern Ireland	Intervention: 16 NHs Control: 16 NHs	Education	Education: 2 h session at baseline, and at 3 and 6 months, Audits Control: usual practice	01/2007–08/2008	MDRO incidence Infection control audit scores	MRSA prevalence was not significantly different between intervention and control groups Infection control audit scores were significantly higher in intervention group compared with control group at 12 months	Medium
Freeman-Jobson et al. (2016) [20]	Quasi-experimental	3 LTCFs in the USA	42 care workers	Education	Education program (three sections]	Not reported	Knowledge related to UTIs	Knowledge scores improved significantly	Low
Fendler et al. (2002) [21]	Quasi-experimental	1 NH in the USA	275 beds	Hand hygiene	Hand sanitizer provided to 2^nd^ and 3^rd^ floors of facility, remainder of facility served as control and received no hand sanitizer	Not reported	Nosocomial infection rates	Reduction in nosocomial infection rates seen in hand sanitizer group	Medium
Ho et al. (2012) [22]	cRCT	18 LTCFs in Hong Kong	Intervention 1: 6 LTCFs Intervention 2: 6 LTCFs Control: 6 LTCFs	Hand hygiene	WHO multi-modal HH interventions: ABHR, gloves, posters, reminders, video clips and performance feedback Intervention 1: slightly powdered gloves Intervention 2: powderless gloves Control: a 2 h health talk	Not reported	HH adherence, infection rates, MDRO incidence	HH adherence was increased after intervention in intervention groups Risks of respiratory outbreaks and MRSA infections requiring hospitalization were reduced in the intervention group	Low
Lai et al. (2019) [23]	Cohort study	11 NHs in Taiwan	11 NHs	Hand hygiene	Education	01/2015–12/2016	Knowledge	Increase in hand hygiene compliance rate, overall knowledge level and use of alcohol-based hand rub	Low
Mody et al. (2003) [24]	Quasi-experimental	2 NHs units in the USA	2 NHs	Hand hygiene	Educational campaign to introduce alcohol based hand rubs	Not reported	Nosocomial infection rates	No difference in nosocomial infection rates after introduction of alcohol based hand rubs	Medium
Schweon et al. (2013) [25]	Quasi-experimental	1 NH in the USA	1 NH	Hand hygiene	HH programme, provision of HH product and wipes, HH education for HCW and patients, Poster as reminder, HH champion, HH compliance monitoring	05/2009–02/2011	Infection rates, MDRO incidence	Significant reduction in LRTIs as well as a non-significant reduction in SSTIs Incidence rates of MRSA, VRE,CDI and gastrointestinal illness were not significantly reduced post-intervention	Low
Teesing et al. (2021) [26]	cRCT	66 units in 33 NHs in the Netherlands	Intervention: 976 beds Control:886 beds	Hand hygiene	Multimodal intervention including a combination of activities for changing hygiene policy and the individual behavior of nurses, E-learning, 3 live lessons, posters, and a photo competition, hand hygiene compliance measurements	10/2016–10/2017	Infection rates, MDRO incidence	Significantly more gastroenteritis and significantly less influenza-like illness in the intervention arm No significant differences of pneumonia, urinary tract infections, and MRSA infections in the intervention arm compared to the control arm	Medium
Temime et al. (2018) [27]	cRCT	26 NHs in France	Intervention: 13 NHs Control: 13 NHs	Hand hygiene	Bundle of HH-related measures: increased availability of alcohol-based handrub, HH promotion, staff education, and local work groups	04/2014–04/2015	Primary: infection rates Secondary: mortality	No data for primary endpoint The intervention group showed significantly lower mortality	Medium
Yeung et al. (2011) [28]	cRCT	6 LTCFs in Hong Kong	Intervention: 3 LTCFs (73 staff, 244 residents) Control: 3 LTCFs (115 staff, 379 residents)	Hand hygiene	Pocket-sized containers of ABHR, a 2-h seminar, reminder materials and posters Control: basic life support education and workshops and usual HH practices	01/2007–11/2007	HH adherence, infection rates	Increase in HH adherence and reduction of the incidence of infections	Low
Banks M et al. (2021) [29]	Quasi-experimental	1 LTCF in the USA	180 beds	Hand Hygiene	HH technology, badge measures alcohol concentration on health care workers hands, or time washing hands	2017–2019	HH adherence, CDI rates	Increase in compliance with hand hygiene, reduction of CDI rate	Low
Sassi et al. (2015) [30]	Quasi-experimental	1 LTCF in the USA	Fomites Before: 106 samples After: 105 samples Staff hands Before: 28 samples After: 29 samples	Hand hygiene	Training: active ingredients, safety precautions, effective times, recommended times to use the product and recommended methods, Product placement: hand sanitizer, wipes, antiviral tissue and gloves	Not reported	MDRO incidence	There was a 16.7% reduction in the number of MS-2 positive, significant reduction in recovered MS-2 on sampled fomites and staff hands	Low
Peterson et al. (2016) [18]	cRCT	12 nursing units at 3 LTCFs in the USA	Between 850—900 beds	IPC Bundle	Universal decolonization for MRSA, active surveillance (all admissions), annual instruction on HH, enhanced cleaning of surfaces (every 4 months)	03/2011–03/2013	MRSA incidence	Significant reduction in rate difference between intervention group and control group	Low
Bellini et al. (2015) [31]	cRCT	104 NHs in Switzerland	Intervention: 53 NHs (2338 residents) Control: 51 NHs (2412 residents)	IPC Bundle	Universal MRSA screening, topical decolonization of carriers, disinfection of environment, standard precautions and training sessions Control: standard precautions alone	06/2010–12/2011	MRSA incidence	No significant reduction in prevalence of MRSA carriers	High
Koo et al. (2016) [32]	cRCT	12 NHs in the USA	Intervention: 6 NHs Control: 6 NHs	IPC Bundle	Interactive educational program: Pre-emptive barrier precautions with gloves and gown, monthly MDRO and infection surveillance with feedback, NH staff education Control: own IPC practices and given knowledge tests	Not reported	Knowledge about IPC topics	Knowledge scores increased significantly after each educational module	Medium
Mody et al. (2015) [33]	cRCT	12 NHs in the USA	Intervention: 6 NHs Control: 6 NHs	IPC Bundle	Pre-emptive barrier precaution, active surveillance for MDROs and infections with feedback, NH staff education on IPC practices and HH promotion Control: own IPC practices	Not reported	MDRO incidence	Intervention group had a significant decrease in overall MDRO prevalence, and lower rates of MRSA acquisition and first new CAUTI	High
McConeghy et al. (2017) [34]	cRCT	5 NHs in the USA	481 and 380 long-stay residents	IPC Bundle	Education, cleaning products, and audit of compliance and feedback	10/2015–05/2016	Infection rates	No significant reduction for both total infections and LRTIs	Medium
Mody et al. (2021) [35]	cRCT	6 NHs in the USA	Intervention: 113 patients Control: 132 patients	IPC Bundle	Enhanced barrier precautions, chlorhexidine bathing, MDRO surveillance, environmental cleaning, education and feedback, hand hygiene promotion	09/2016–08/2018	MDRO incidence	Reduced overall prevalence of MDRO	Medium
Ben-David et al. (2019) [36]	Quasi-experimental	330 LTCFs in Israel	330 LTCFs	IPC Bundle	Education, screening, isolation	2009–2015	MDRO incidence	Incidence of MDRO acquisition declined in all facility types to approximately 50% from baseline	Low
Trick et al. (2004) [37]	cRCT	1 skilled NH in the USA	283 residents	Isolation	Healthcare workers assigned to either the contact isolation group or routine glove use group without contact isolation	06/1998–12/1999	MDRO incidence	No difference in acquisition of VRE/MRSA with glove use without contact isolation compared to contact isolation group	High
Adachi et al. (2002) [38]	RCT	2 NHs in Japan	141 residents	Oral hygiene	Professional oral care weekly by dental hygienists in intervention group, usual care in control group	Not reported	Oral health	Professional oral care by dental hygienist reduced microorganisms related to pneumonia	Low
Ishikawa et al. (2008) [39]	Quasi-experimental	3 NHs in Japan	202 residents	Oral hygiene	Provided professional oral care by a dental hygienist once a week with varying modality, intensity and frequency	Not reported	Oral health	Levels of oropharyngeal bacteria decreased across all 3 facilities when weekly professional care was instituted	Low
Kulberg et al. (2010) [40]	Quasi-experimental	1 NH in Sweden	43 residents	Oral hygiene	Dental hygiene education led by dental hygienist for nursing staff; residents were given electronic toothbrushes,recommended to use chlorhexidine gel twice daily	2008	Oral health	Reduction in plaque scores	Low
Maeda and Akagi (2014) [41]	Cohort study	1 LTCF in Japan	Intervention: 31 residents Control: 32 residents	Oral hygiene	Oral care protocol (at least twice per day), tooth and tongue brushing using a toothbrush, and oral mucosa brushing using a sponge brush and a 0.2% chlorhexidine solution, moisturizing the inner mouth with glyceryl poly methacrylate gel, salivary gland massage Control: oral care not performed regularly	07/2011–06/2013	Pneumonia rates	Reduction in the incidence of pneumonia	Medium
Quagliarello et al. (2009) [42]	RCT	1 LTCF in the USA	52 residents (30 in oral hygiene intervention group, 20 in swallowing intervention group)	Oral hygiene	Oral hygiene group assigned to manual oral brushing plus chlorhexidine mouth rinse at different frequencies daily, no control Swallowing group assigned to 90 degree feeding posture, swallowing techniques or manual brushing daily	Not reported	Oral health	Significant reduction in plaque scores at end of oral care intervention	Medium
Yoneyama et al. (2002) [43]	RCT	11 NHs in Taiwan	417 residents	Oral hygiene	Enforced oral hygiene measures and oral cleaning by dental hygienists once a week, control group received usual care	1996–1998	Pneumonia rates	Incidence of pneumonia was lower in intervention group	Medium
Cabezas et al. (2021) [44]	Cohort study	NH in Spain	28.000 residents, 26.000 NH Staff, 60.000 HCW	Vaccination	Participants (NH-Residents, NH-staff and HCW) were followed until outcome (SARS-Cov2 infection, hospital admission, death) occurs, vaccination as a time varying exposure	12/2020–05/2021	SARS-CoV-2 infection rates, hospital admission or death with Covid-19	Vaccination was associated with 80–91% reductions in symptomatic and asymptomatic SARS-CoV-2 infections among nursing home residents, nursing home staff, and healthcare workers and led to ≥ 95% reductions in covid-19 related hospital admission and mortality among nursing home residents	Low
Goldin et al. (2022) [45]	Cohort study	454 LTCFs in Israel	43.596 residents	Vaccination	BNT162b2 mRNA COVID-19 (Comirnaty) Vaccine	12/2020–05/2021	SARS-CoV-2 infection rates	Mortality from COVID-19 was 21.9% in the vaccinated population and 30.6% in the unvaccinated population	Medium
Maruyama et al. (2010) [46]	RCT	9 hospitals and 23 NHs in Japan	1006 residents	Vaccination	Residents received pneumococcal vaccine, control group received placebo	03/2006–03/2009	Pneumonia rates	Significant reduction of pneumonia incidence	High

LTCF, long-term care facilities; MDRO, multi-drug resistant Organism; CDI, C.difficile Infection; CDC, Centers for Disease Control and Prevention; RD, resident days; DOT, days of therapy; AIRR = ; UTI, urinary tract infection; RCT, randomized-control trial; cRCT, cluster randomized-control trial; NH, Nursing Home; MRSA, methicillin-resistant Staphylococcus aureus; WHO, World Health Organization; ABHR, alcohol-based hand rub; HH, hand hygiene; HCW, healthcare worker; LTRI, lower respiratory tract infection; SSTI, skin and soft tissue infection; VRE, Vancomycin-resistant Enterococci; IPC, infection prevention and control; CAUTI, Catheter-associated urinary tract infection; CRE, Carbapenem-resistant enterobacteriaceae

**Table 2:** Included studies from the outbreak setting

Author	Design	Setting	Pathogen or disease	Sample size	Topic	N of cases	Overall attack rate	Outbreak Date	Control measures	Results	Mean quality score
Ahmed et al. (2018) [47]	Case–control study	1 LTCF in the USA	GAS	228-bed skilled nursing facility	IPC bundle	7 residents and 5 staff	0.84% resident: 0.65% Staff: 1.41%	05/2014–08/2016	Active surveillance, contact precaution, recommendation for use of PPE during irrigation, changing soiled diapers/linen before dressing change, and adopting a supportive sick leave policy	Frequent antimicrobial treatment and wound vacuum-assisted closure devices as risk factors	Medium
Al Hamad et al. (2021) [48]	Outbreak report	1 LTCF in Qatar	Covid-19	Not reported	IPC bundle	24 cases	Not reported	06/2020	Education, awareness, staff compliance monitoring, contact tracing, visitor policy revision, monitoring	Lapse of infection control practices, successful containment of the outbreak, only 57% of patients were symptomatic	Low
Barret et al. (2014) [49]	Outbreak report	1040 LTCFs in France	Gastroenteritis (Norovirus 73%, Rotavirus 19%)	Residents and staff	IPC bundle	26.551 episodes among resident, 5.548 episodes among staff	resident: 32.5% Staff: 12.40%	05/2010–05/2012	Reinforcement of hand hygiene, contact precautions, cleaning or disinfection of the environment, restriction of movements, stopping or limitation of group activities, measures on food handling	The attack rate was lower and the duration of outbreaks was shorter when infection control measures were implemented within three days of onset of the first case	Low
Bernadou et al. (2021) [50]	Outbreak report	1 NH France	Covid-19	88 residents, 104 staff	IPC bundle	109 cases	55%	03–05/2020	Mass testing, symptom screening, active surveillance, droplet measures	Significant rate of asymptomatic residents detected through mass screening	Low
Bruins et al. (2020) [51]	Outbreak report	1 NH in the Netherlands	MDRO	110 residents	IPC bundle	8 cases	7%	02/2017–05/2018	Screening, contact precautions, intensive cleaning procedure, education	Spread was associated with the use of shared toilets in communal areas. Containment of the outbreak after the implementation of a customized IPC bundle	Low
Calles et al. (2017) [52]	Case–control study	1 LTCF in the USA	Hepatitis C	114-bed skilled nursing facility	IPC bundle	All cases: 45 residents, case–control: 30 cases/ 62 controls	Overall: 10.54%, Residents:15.63% Staff: 0%	01/2011–09/2013	Screening, environmental measures, use of single-use of instruments, cleaning and disinfection, glove change,	Podiatry care and INR monitoring by phlebotomy were significantly associated with HCV cases	Medium
Dom´ınguez- Berjo´n et al. (2007) [53]	Cohort study	1 NH in Spain	Adenovirus	118 residents	IPC bundle	46 cases (36 residents and 10 HCWs)/193 controls	Overall: 19.25%, Resident: 30.51% Staff: 8.26%	08–12/2005	Cleaning and disinfection, hand hygiene, isolation, withdrawal of affected workers, admission stop, visitor restrictions, education	Age, nursing home floor and cognitive impairment as independent risk factors	Medium
Dooling et al. (2013) [54]	Case–control study	1 LTCF in the USA	GAS	Not reported	IPC bundle	Total: 19 residents with 24 infections Case- control study: 18 infections/54 controls	Not reported	06/2009–06/2012	Carriage survey, contact precaution, education, and placement of additional alcohol-based hand rub dispensers, cleaning and disinfection, chemoprophylaxis	Indwelling line and area of living as independent risk factors	Medium
Gaillat et al. (2008) [55]	Outbreak report	1 NH in France	ILI (Influenza A)	81 residents	IPC bundle	32 residents and 6 staff	Overall 29.46%, Residents: 39.51% Staff: 12.50%	06–07/2005	Isolation, wearing of surgical masks, droplet and contact precaution, chemoprophylaxis, setting up a crisis management team	This outbreak occurred in summer Spread of the virus because of close area of living	Low
Hand et al. (2018) [56]	Outbreak report	1 LTCF in the USA	Coronavirus NL63	130 residents	IPC bundle	20 cases	26%	11/2017	Standard and droplet precaution, hand hygiene, enhanced environmental cleaning	Outbreak report with Coronavirus NL63	Low
Kanayama et al. (2016) [57]	Case–control study	1 LTCF in Japan	MRPA	Residents in a 225-bed LTCF	IPC bundle	Total: 23 cases Case- control study: 14 cases/28 controls	Not reported	01/2013–01/2014	Surveillance, infection control team composition, contact precautions, cohorting and using new gloves and gown, admission restriction, training and re-education of HCWs, deep environmental cleaning, discontinuation of sharing devices	Use of an oxygen mask and use of a nasogastric tube were significant factors associated with MRPA infection	Low
Mahmud et al. (2013) [58]	Multiple outbreak reports	37 LTCFs in Canada	Influenza A (47%), Influenza B (5%), Parainfluenza (5%), Respiratory syncytial virus (3%), not identified (40%)	Residents and staff in 37 LTCFs	IPC bundle	154 outbreaks	Median (Influenza A and B) residents: 7.2%, staff: 3.3%	Median: 18 days (3–53 days)	Chemoprophylaxis: 57% of influenza A, 63% of influenza B (the other measures were not reported), early notification	Early notification to public health authorities was associated with lower attack rate and mortality rates among residents, Chemoprophylaxis was the measure associated with lower attack rates, but not with shorter duration of outbreaks or with lower mortality	Low
McMichael et al. (2020) [59]	Outbreak report	1 LTCF in the USA	COVID-19	130 residents and 170 staff	IPC bundle	167 cases (101 residents, 50 HCW, 16 visitors)	Residents: 77.6% HCW: 29.4%	02–03/2020	Case investigation, contact tracing, quarantine of exposed persons, isolation, on-site enhancement of IPC measures	Outbreak description of one of the first COVID-19 outbreaks in a LTCF	Low
Murti et al. (2021) [60]	Outbreak report	1 NH in Canada	COVID-19	65 residents	IPC bundle	Residents: 61, Staff: 34	Residents: Attack rate 94%, case fatality rate 45%; Staff: Attack rate 51%	03–05/2020	Droplet and contact precautions, universal masking of staff, testing, visitor restrictions	Tight clustering of cases with high attack rate of 94%, Outbreak containment after IPC implementation	Low
Nanduri et al. (2019) [61]	Outbreak report	1 LTCF in the USA	GAS	Not reported	IPC bundle	19 invasive and 60 non-invasive cases (50 residents and 24 staff)	Not reported	05/2014–08/2016	Chemoprophylaxis, active surveillance, recommendation of health authority	Inadequate infection control and wound-care practices may lead to this prolonged GAS outbreak in a skilled nursing facility	Low
Nicolay et al. (2018) [62]	Outbreak report	1 NH in France	Acute gastroenteritis (Norovirus)	Nursing home with 89 residents	IPC bundle	29 residents and 9 staff	43.94% Resident: 57.65% Staff: 19.15%	09–10/2016	Reinforcement of standard precaution, barrier measures, limitation of the movements of symptomatic residents, environmental disinfection, stopping group activities, closure of the kitchen and outsourcing of meals	More dependent residents were at higher risk of acute gastroenteritis	Low
Psevdos et al. (2021) [63]	Outbreak report	1 NH in the USA	COVID-19	80 residents	IPC bundle	25 residents	Attack rate 31%, mortality rate 24%	03–04/2020	Testing, visitor restrictions, symptom screening, admission stop, hand hygiene, masks, isolation,	Attack rate only 31%. Quick containment of the outbreak through IP measures	Low
Sáez-López et al. (2019) [64]	Outbreak report	1 LTCF in Portugal	Norovirus	335 residents	IPC bundle	146 people, 97 residents and 49 staff	Residents: 29%, Nurses: 19%	10–12/2017	Disinfection, hand hygiene, education, PPE, isolation and cohorting	Insufficient adherence to IPC measures due to staffing shortage	Low
Shrader et al. (2021) [65]	Outbreak report	1 LTCF in USA	COVID-19	98 residents, 156 staff	IPC bundle	52 residents and 19 staff	Resident 52%	03–08/2020	Testing, PPE, disinfection and isolation, restriction of visitors	Outbreak controlled with IPC measures	Low
Telford et al. (2021) [66]	Observational study	24 LTCF in the USA	COVID-19	2580 LTCF residents	IPC bundle	1004	39%	06–07/2020	Adherence to IPC (HH, Disinfection, Social Distancing, PPE, Symptom screening)	Implementation lowest in Disinfection, highest in symptoms screening, differences in social distancing and PPE between high-prevalence and low-prevalence group	Medium
Thigpen et al. (2007) [67]	Outbreak report	1 NH in the USA	GAS	Residents in a 146-bed nursing home	IPC bundle	Definite case: 6 residents Possible case: 4 residents	6.9%	11–12/2003	Screening, reinforce standard precautions, improve access to hand disinfectants, to implement appropriate respiratory etiquette, influenza immunization, Chemoprophylaxis for colonized persons	Three risk factors for GAS: presence of congestive heart failure or history of myocardial infarction, residence on unit 2, and requiring a bed bath	Low
Van Dort et al. (2007) [68]	Case–control study	1 NH in the USA	NTHi	120-bed nursing home	IPC bundle	13 cases 18 controls	Not reported	06–07/2005	Universal precaution, respiratory droplet precaution, evaluating staffs with symptoms, throat culture survey for residents	None of the variables showed a significant association with NTHi	Medium
Van Esch et al. (2015) [69]	Case control study, Outbreak report	1 LTCF in Belgium	CDI	120 bed LTCF	IPC bundle	66 cases 61 controls	51.97%	01/2009–12/2012	Stringent hygienic protocol, active surveillance, strict isolation, timely treatment for CDI (AB-prescription), cleaning and disinfection of residents rooms	The nutritional status was found to be significantly poorer in the residents with CDI	Low
Weterings et al. (2015) [70]	Outbreak report	1 hospital and 1 NH in the Netherlands	KPC-KP	150-bed nursing home	IPC bundle	4 cases	Not reported	07–12/2013	Isolation, PPE, Handrub with 70% alcohol, frequent audits of hand hygiene and direct feedback, daily cleaning of room and disinfection, contact screening surveillance	Preventing transmission of MDROs is challenging in nursing homes	Low
Kennelly et al. (2021) [71]	Observational study	45 NH in Ireland	COVID-19	2043 residents	Surveillance	1741 cases	43.9%, 27.2% asymptomatic, fatality rate 27.6%	04–05/2020	Surveillance	Significant impact of Covid-19 with high rate of asymptomatic carriers	Low
Blackman et al. (2020) [72]	Outbreak report	1 NH in the USA	COVID-19	150 bed institution	Testing	32 symptomatic residents, 26 HCW, limited testing capacity	Not reported	Not reported	Education, personal protective equipment, masks, symptom screening, contact and droplet precautions	Severe outbreak despite IPC measures because of insufficient testing availability	Low
Dora et al. (2020) [73]	Outbreak report	1 NH in the USA	COVID-19	99 residents	Testing	Residents:19 Staff: 8	Residents: 19%; Staff 6%	03–04/2020	Screening, droplet and contact precautions, visitor restrictions	Successful outbreak containment	Low
Eckardt et al. (2020) [74]	Outbreak report	1 LTCF in the USA	COVID-19	120 bed LTCF	Testing	Not reported	5.4%, 3.6% and 0.41% in three point prevalence testing rounds every 14 days	Not reported	Point prevalence testing	Containment of outbreak	Low
Graham et al. (2020) [75]	Outbreak report	4 NH in the UK	COVID-19	394 residents and 70 staff	Testing	Residents: 126 Staff: 3	40% with 43% asymptomatic, 26% mortality	03–05/2020	Two point prevalence surveys	60% of SARS-CoV-2 positive residents were either asymptomatic or only had atypical symptoms for Covid-19	Low
Louie et al. (2021) [76]	Outbreak report	4 LTCF in the USA	COVID-19	431 persons	Testing	214	49.7%; thereof 40.2% asymptomatic	03–04/2020	Surveillance	Mass testing identified a high proportion of asymptomatic infections	Low
Patel et al. (2020) [77]	Cohort study	1 LTCF in the USA	COVID-19	127 residents	Testing	33 thereof 13 asymptomatic	26%	Not reported	Surveillance	High rate of asymptomatic infections	Medium
Roxby et al. (2020) [78]	Outbreak report	1 LTCF in the USA	COVID-19	80 residents and 62 HCW	Testing	3 residents, 2 staff	3.8% of residents, 3.2% of staff	Not reported	Mass testing, symptom screening	Detection of asymptomatic infected residents	Low
Sacco et al. (2020) [79]	Outbreak report	1 LTCF in France	COVID-19	87 residents and 92 staff members	Testing	41 residents and 22 staff members	47% in residents and 24% in staff	03–04/2020	Mass testing	High rate of asymptomatic infected persons	Low
Sanchez et al. (2020) [80]	Outbreak report	26 LTCF in the USA	COVID-19	2773 residents	Testing	1207 cases	44%	03–05/2020	Mass testing (two point-prevalence surveys)	44% attack rate, 37% hospitalization, 24% mortality; Reduction of positivity after second point prevalence survey	Low
Zollner et al. (2021) [81]	Outbreak report	3 LTCF in Austria	COVID-19	277 residents	Testing	36	13%	03–04/2020	Testing	Only 25% with fever and 19% with cough, 6/36 remained asymptomatic, hospitalization rate 58% and mortality rate 33%,19/214 HCW positive	Low
Giddings et al. (2021) [82]	Cohort study	330 LTCF in UK	COVID-19	Resident and staffs	Vaccination	297 outbreaks across all four time periods	90% of LTCF	Not reported	Vaccination	Reduction of number of the proportion of LTCF with outbreaks over the four time periods from 51.5% to 4.7%	Medium
Martinot et al. (2021) [83]	Outbreak report	1 LTCF in France	COVID-19	93 residents	Vaccination	40 cases (residents 24, HCW 16)	Residents 25.8%, HCW 21.9%	03–05/2021	Vaccination	Outbreak with alpha-variant,higher case rate in unvaccinated than in vaccinated residents, no severe symptoms in vaccinated residents	Low
Mazagatos et al. (2021) [84]	Outbreak report	LTCFs in Spain	COVID-19	Not reported	Vaccination	Not reported	Not reported	12/2020–04/2021	Vaccination	Effectiveness of 71%, 88% and 97% for infections, hospitalization and death	Low
Van Ewijk et al. (2022) [85]	Outbreak report	1 LTCF in the Netherlands	COVID-19	105 residents	Vaccination	70 residents	67% (70/105)	11/2021–01/2022	Booster vaccine dose	Booster vaccine curbed transmission	Low
Cheng H-Y et al. (2018) [86]	Outbreak report	LTCFs in Taiwan	Influenza	102 Outbreaks	Vaccination, antiviral treatment/prophylaxis	Median residents 65.5	Median attack rate 24%	2008–2014	Antiviral prophylaxis	Initiating antiviral treatment within 2 days of outbreak start decreased the possibility of a large influenza outbreak to only one-third	Low

LTCF, Long-term care facilities; GAS, Group A Streptococcus; IPC, Infection prevention and control; NH, Nursing Home; MDRO, multi-drug resistant Organism; INR, International Normalized Ratio; HCV, HepatitisCVirus; OR, Odds ratio; CI, Confidence interval; ILI, Influenza-like-illness; HCW, Healthcare worker; aOR, adjusted Odds ratio; RR, Risk ratio; HH, Hand hygiene; PPE, Personal Protective Equipment; NTHi, Non-typeable Haemophilus influenzae; CDI, C.difficile Infection; AB, Antibiotic; KPC-KP, Carbapenemase-producing Klebsiella pneumoniae

Details for study type, study quality, place of study, and type of intervention are summarized in Table 3.

**Table 3:** Characteristics of the included studies with respect of study quality,-type,-place and type of intervention

Variable	Total (%)	Non-outbreak (%)	Outbreak (%)
Studies	74 (100)	34 (46)	40 (54)
*Study quality*			
High quality	4 (5)	4 (100)	0
Medium quality	22 (29)	14 (64)	8 (36)
Low quality	48 (65)	16 (33)	32(67)
*Study type*			
RCT	18 (24)	18 (100)	0
Cohort study	10 (13)	7 (70)	3 (30)
Case control study	6 (8)	0	6 (100)
Outbreak report	30 (41)	0	30 (100)
Others (single arm trial, before-after study)	10 (13)	9 (90)	1 (10)
*Place of study*			
Europe	23 (31)	6 (26)	17 (74)
North America	38 (51)	18 (47)	20 (53)
Asia	13 (18)	10 (77)	3 (23)
*Type of intervention*			
IPC bundle	31 (42)	7 (23)	24 (77)
Mass testing	11 (15)	0	11 (100)
Hand hygiene	10 (14)	10 (100)	0
Education	2 (3)	2 (100)	0
Isolation precautions	1 (1)	1 (100)	0
Oral hygiene	6 (8)	6 (100)	0
Vaccination	7 (9)	3 (43)	4 (57)
Decolonization	1 (1)	1 (100)	0
Antimicrobial stewardship	4 (5)	4 (100)	0
Antiviral prophylaxis	1 (1)	0	1 (100)
*Measured outcomes*			
Infection rates	33 (45)	14 (42)	19 (58)
MDRO incidence	12 (16)	11 (92)	1 (8)
Oral health	4 (5)	4 (100)	0
Adherence to IPC measures	3 (4)	3 (100)	0
Knowledge about IPC topics	2 (3)	2 (100)	0
Outbreak control	8 (11)	0	8 (100)
Risk factor identification	12 (16)	0	12 (100)

RCT, randomized controlled trial; IPC, infection prevention and control; MDRO, multi drugresistant organism

### Type of intervention and setting

**Fig. 2:**
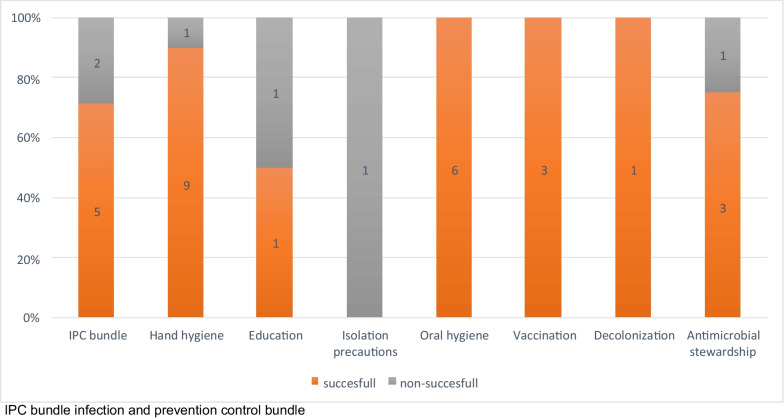
Non-outbreak setting, divided in successful and non-successful intervention by type of intervention

The most frequent interventions from the non-outbreak setting were hand hygiene (N = 10) [21–30], an IPC bundle with several measures included (N = 7) [18, 31–36], oral hygiene (N = 6) [38–43], antimicrobial stewardship (N = 4) [13–16] as well as vaccination of residents (N = 3) [44–46]. Interestingly, studies from Asia mainly concentrated on oral health (N = 4) [38, 39, 41, 43] and hand hygiene (N = 3) [22, 23, 28], whereas studies from North America drew their attention towards antimicrobial stewardship [13–16] and hand hygiene [21, 24, 25, 30] (each N = 4). An overview on the results of the included studies in non-outbreak settings is shown in Fig. 2.

The majority of studies in the outbreak setting concentrated on an IPC bundle (N = 24) [47–70] and on mass testing/surveillance (N = 11) [71–81].

#### Hand hygiene

Hand hygiene alone was evaluated in ten studies [21–30], all conducted in non-outbreak settings. Nine of ten articles showed a successful intervention with reduced infection rates and lower prevalence of multi drug resistant organisms (MDRO) [21–23, 25–30].

No study evaluated hand hygiene alone in an outbreak setting.

#### Antimicrobial stewardship

Four studies in non-outbreak-settings on antimicrobial stewardship which also measured the infection rates were included in our review [13–16]. Three could demonstrate a reduction of *C.difficile* infections through antimicrobial stewardship [14–16], while one retrospective quasi-experimental study showed no decrease of MDRO-incidence or *C.difficile* infections [13].

In an outbreak setting no studies on this topic were undertaken so far.

#### Education

Two studies assessed the effect of education in IPC measures [19, 20]. Both were executed in a non-outbreak setting. One RCT found no difference of methicillin-resistant Staphylococcus aureus (MRSA) prevalence in groups with IPC education [19]. The other study recorded a successful outcome with a significant improvement of knowledge after education [20].

No studies were conducted to evaluate the effect of education alone in an outbreak setting.

#### Decolonization

One RCT assessed decolonization measures as main intervention in a non-outbreak setting [17] and found a reduction of MRSA prevalence after decolonization measures were implemented. No study evaluated decolonization measures in an outbreak setting.

#### Isolation precautions

One high-quality study from the USA evaluated the effect of isolation precautions alone with no significant difference in MDRO prevalence with/without isolation precautions [37].

#### Vaccination

We included three studies on vaccination in a non-outbreak setting [44–46]. A high-quality trial from Japan showed a significant reduction in cases of pneumonia in residents of 23 LTCF after the 23-valent pneumococcal vaccine was introduced [46]. Two studies were conducted in the non-outbreak setting with COVID-19 vaccination and showed a significant reduction in COVID-19 cases, COVID-19 related hospitalization and mortality [44, 45]. In outbreak settings, COVID-19 vaccination of residents significantly reduced outbreaks, COVID-19 cases, COVID-19 related hospitalization, and mortality in 3 of 4 studies. One study, executed in the turn of the year 2021 to 2022 showed no reduction in COVID-19 cases, but a reduced case fatality after vaccination [85].

#### Oral hygiene

Six studies evaluated the effect of improved oral hygiene on overall infection rates, all from a non-outbreak setting [38–43]. All studies found a reduction of infections (mainly cases of pneumonia) with the intervention.

No publication on the effect of oral hygiene in an outbreak setting was recorded.

#### Mass testing

We found no study on mass testing in a non-outbreak setting. All studies that analyzed the effect of mass testing were performed in an outbreak setting during an early stage of the COVID-19 pandemic [72–81] and mostly resulted in the isolation of residents and quarantine of HCWs who tested positive. All of them found a significant number of asymptomatic HCWs and residents with a range of asymptomatic carriers from around 3% up to 43% in different studies.

#### IPC bundles

Half of the included studies (21% in non-outbreak-setting [18, 31–36] and 60% in outbreak setting [47–70]) focused on several topics simultaneously within an IPC bundle. In the non-outbreak setting one cRCT study evaluated a bundle of education of health care workers (HCW), surface cleaning, and feedback on HAI rates and could not observe a significant reduction in infection rates [34].

Furthermore, a large RCT in 104 long-term care facilities in Switzerland showed no effect of MRSA decolonization and different isolation precautions (standard vs. contact precautions) on MRSA prevalence [31].

In contrast, four studies could demonstrate a reduction of MDRO prevalence through a multicomponent intervention that included barrier precautions, active surveillance of MDRO and infections, as well as staff education and hand hygiene promotion [18, 33, 35, 36]. Koo et al. could at least show an improvement in knowledge for trained topics through an IPC bundle that included education while not evaluating infection rates [32]. Twenty-four of 31 included studies on IPC bundles were performed in an outbreak setting [47–70]. The included studies contained cohort and case–control studies, as well as outbreak reports. A median of 5 measures were included in an IPC bundle (range 2 to 8) with isolation/precautions (N = 24, 19.7%), surveillance (N = 13, 10.7%) and hand hygiene (N = 9, 8.2%) being the most represented interventions included in the bundles. All outbreak reports showed containment of the outbreaks.

When we differentiated by the transmission route, we found 15 studies where the transmission occurred mainly by respiratory droplets (SARS-CoV-2, Group A streptococci, Influenza-like illnesses) [47, 48, 50, 54–56, 58–61, 63, 65–68] and 8 studies with transmission via direct and/or indirect contact (gastroenteritis, MDRO, Norovirus etc.) [49, 51, 53, 57, 62, 64, 69, 70]. The bundles in these two categories varied slightly. The ones for pathogens transmitted through the respiratory route concentrated on wearing masks and repetitive testing, whereas those for direct or indirect contact transmissions focused more on environmental cleaning measures and contact precautions.

#### COVID-19

In the non-outbreak setting we found two articles focusing on the effect of vaccination on SARS-CoV-2 infection rates [44, 45]. Both found a positive effect of the vaccination on infection incidence in nursing home residents and staff as well as a reduced mortality in residents.

In 22/40 (55%) studies from the outbreak setting, SARS-CoV-2 was the main pathogen [48, 50, 59, 60, 63, 65, 66, 71–85]. Vaccination was also highly effective in reducing infections in this setting [82–85]. 7 articles reported the effect of an IPC bundle [48, 50, 59, 60, 63, 65, 66], whereas mass testing was the main IPC measure in 11 articles [71–81] (see also paragraph on mass testing above) and vaccination was evaluated in four studies [82–85]. As already mentioned above, most of the included studies from the outbreak setting documented a successful containment of the outbreak. This was also true for COVID-19.

#### Other WHO core components

Other WHO core components for infection prevention, such as IPC programs per se, IPC guidelines, monitoring of IPC practices, reduction of workload, optimized staffing and bed occupancy rates as well as the environment, materials and equipment alone were not evaluated in the studies that were identified by our search.

### Study quality

The quality of included studies was generally low (Additional file 1: Tables S2a, S2b, S2c). Only four (5%) studies were classified as high quality [31, 33, 37, 46]; all of these were RCTs. Other RCTs were medium (N = 10) [15, 17, 19, 26, 27, 32, 34, 35, 42, 43] or low (N = 4) in quality [18, 22, 28, 38]. In contrast, the included cohort studies were medium-quality [21, 24, 41, 53, 82] or low-quality studies (N = 5) [29, 36, 39, 44, 77]. The case–control studies were classified as medium (N = 4) [47, 52, 54, 68] or low quality (N = 2) [57, 69]. All outbreak reports were classified as low quality per definition (N = 16) [48–51, 55, 56, 58–65, 67, 70].

## Discussion

### Main results

In this systematic review, which also covers the SARS-CoV-2 pandemic, we identified 74 studies of different quality evaluating the effect of infection prevention and control measures in long-term care facilities in outbreak or non-outbreak settings, respectively. Hand hygiene, staff education measures, antimicrobial stewardship, vaccination and oral care seem to be consistently effective in preventing healthcare-associated infections or transmission events in long-term care settings. However, studies were mostly of low quality and highly heterogeneous with regard to setting, intervention measures, populations, and outcomes. Therefore, deriving standard of care recommendations or guidelines for LTCFs based on these data remains difficult.

Our current systematic review covers data from non-outbreak and outbreak settings, especially during the SARS-CoV-2 pandemic, from a variety of countries worldwide. With a large increase in new publications during the COVID-19 pandemic, our study provides an update on the currently available literature on the effectiveness of different infection prevention measures in LTCFs in comparison to previous reviews. This allowed us to draw a more accurate picture of the current evidence on this topic.

For non-outbreak publications, our results regarding the effectiveness of different measures as well as the difficult comparability of the studies are in line with earlier well-made systematic reviews [7, 87]. In comparison to Lee et al., we identified relatively good quality data on the importance of hand hygiene, antimicrobial stewardship, vaccination and oral hygiene in addition to the already known beneficial effects of education, monitoring and multi-modal strategies. Of note, Lee et al. did not evaluate any antimicrobial stewardship interventions in their review [7]. While Uchida et al. focused solely on therapeutic measures [87] we also analyzed studies on educational measures and focused more on the effect of the type of intervention. This allowed us to identify the particular contribution of various measures to a given outcome.

In contrast to others authors [7, 87–90], we included articles from the non-outbreak setting as well as from the outbreak-setting. While one review on IPC measures in the outbreak setting was conducted before COVID-19 [90], the others were published during the pandemic [88, 89].

For the outbreak setting, mainly for studies on SARS-CoV-2, our review indicates that reasonably good data exist for the effectiveness of vaccination, mass testing, and IPC bundles, whereas no statement can be made about other single or combination of measures [71, 72]. Since outbreaks in general and virus-related outbreaks in particular are often self-limiting [91], it remains difficult to assess and put into context the added value of such transiently applied outbreak control measures. Whether an outbreak could be contained because of the IPC bundle or because of the temporary nature of outbreaks is impossible to discriminate in studies without control group.

It is to be expected that a combination of different measures produces an additive or synergistic effect, although, in our review, combinations of different measures were mostly applied in outbreak settings, with a difficult to evaluate outcome for the reasons mentioned above. Therefore, an additive or synergistic effect cannot be proven in our dataset.

Although education is often part of a bundle of measures, there is very little data on the importance of education alone. However, this should not limit the importance of education, which is extremely important in this context where health care workers are often insufficiently trained in medical and infection prevention and control.

### Strengths and limitations

Our study has several limitations. First, generalizability is hampered in that we only included studies published in English and most studies in our review were performed in North America and Europe. As long-term settings vary widely within and across countries, settings and thus effectiveness of interventions may differ across institutions. Second, publication bias may have played a role in that ineffective IPC interventions may not be published, especially in outbreak settings. Furthermore, due to the heterogeneity and the low quality of studies, we were unable to compare effect sizes, let alone to meta-analyze effects across studies, even within similar settings or types of interventions. Last, we did not extend our search beyond PubMed and The Cochrane Central Register of Controlled Trials (CENTRAL), but given the quality and heterogeneity of identified studies, we are confident that searching further databases would not have led to more refined results. Another limitation of our study is the fact that LTC institutions provide medical and nursing care for different and rather heterogeneous resident populations in different countries. Thus, an identical measure could have a different clinical outcome based on the cognitive and or functional status of the persons living in the LTCF. This also applies to common geriatric syndromes such as frailty and/or malnutrition including urinary or stool incontinence. In addition the way how and by whom medical care is provided may have some impact upon the outcomes documented in our selected studies.

Strengths of our study are the inclusion of studies conducted in both non-outbreak and outbreak settings, including the COVID-19 pandemic and outbreaks of other pathogens, the inclusion of antimicrobial stewardship as a topic and the updated search until November 2022. Through this, we were able to recognize a large amount of studies with IPC measures not included in other reviews.

### Conclusion and outlook

In conclusion, although we were able to find a good amount of data on IPC measures in the LTCF setting, interpretability and generalizability of these data remains difficult. Especially for outbreak settings, reports of successful control measures often do not add more value than do single case reports in the individual patient care setting. Given that the population at risk for healthcare-associated infections in these settings is large and constantly growing, coordinated action is imperative. In order to move a step forward and to complete the picture, well executed studies on this topic are desperately needed. These include a systematic evaluation of clearly defined single interventions or intervention bundles using high-quality (cluster-)-randomized controlled trials in well-defined settings and patient populations with useful outcome measures. These, due to the special needs of this population, do not only include HAIs, but also other measures such as quality of life, which sometimes might be favored over restrictive measures for infection prevention. In addition, IPC intervention trials and or measures across a clearly defined resident population and interventions that control for geriatric syndromes are urgently needed. Such efforts are only possible if sufficient funding for large, concerted, multi-national initiatives is available.

In general, it can be discussed whether reducing nosocomial infections is of high priority for the long-term-care setting or whether the focus should rather be on maintaining quality of life. Data on the influence of IPC measures on quality of life in long-term-care facilities are scarce or non-existing. From the COVID-19 pandemic, we assume that certain factors, such as visitor restriction, isolation measures and wearing masks for example, had an impact on the well-being of APH residents.

In the meantime, using the available low-quality evidence and extrapolating infection prevention and control measures from acute to long-term care with some common sense seem to be useful approaches. Thereby, the most essential basic IPC measures from the acute care setting, such as standard hygiene measures with hand hygiene and personal protective equipment when needed, combined with a good education for HCW and a functioning surveillance system might be the cornerstones of a successful IPC program in long-term care. Given that LTCFs are very heterogeneous with ever changing activities, defining the needs of every single institution is challenging. However, a standardized IPC program that every institution could adapt to its temporary needs may be a reasonable approach with a high acceptance on the part of the residents, HCW, and IPC team.

### Supplementary Information

**Additional file 1**. Quality assessment of the included studies

## Data Availability

The dataset used and/or analysed during the current study are available from the corresponding author on reasonable request.
