# Supplementary material for: Effective infection prevention and control measures in long-term care facilities in non-outbreak and outbreak settings: a systematic literature review
Source: Antimicrob Resist Infect Control. 2023 Oct 18;12:113. doi: 10.1186/s13756-023-01318-9 (PMC10585745; doi:10.1186/s13756-023-01318-9)
Supplement: Supplementary file 1 — Additional file 1. Quality assessment of the included studies [file 13756_2023_1318_MOESM1_ESM.docx]

**Supplement**

Table S2a) Quality assessment of randomized controlled trials according to the Revised Cochrane risk-of-bias tool for randomized trials.

Table S2b): Quality assessment of cohort studies according to the Newcastle-Ottawa quality assessment scale for cohort studies

Table S2c): Quality assessment of case control studies according to the Newcastle-Ottawa quality assessment scale for case control studies
